# Use of a Physiologically Based Pharmacokinetic Model for Rats to Study the Influence of Body Fat Mass and Induction of CYP1A2 on the Pharmacokinetics of TCDD

**DOI:** 10.1289/ehp.8805

**Published:** 2006-04-18

**Authors:** Claude Emond, Linda S. Birnbaum, Michael J. DeVito

**Affiliations:** 1 National Research Council, National Academy of Sciences, Washington, DC, USA; 2 National Health and Environmental Effects Research Laboratory, U.S. Environmental Protection Agency, Research Triangle Park, North Carolina, USA; 3 Environmental and Occupational Health Department, Medicine Faculty, University of Montreal, Montreal, Quebec, Canada

**Keywords:** adipose tissue, AhR, aryl hydrocarbon receptor, dioxin, modeling, PBPK, pharmacokinetics, TCDD

## Abstract

2,3,7,8-Tetrachlorodibenzo-*p*-dioxin (TCDD) is a highly lipophilic chemical that distributes into adipose tissue, especially at low doses. However, at high doses TCDD sequesters in liver because it induces cytochrome P450 1A2 (CYP1A2) that binds TCDD. A physiologically based pharmacokinetic (PBPK) model was developed that included an inducible elimination rate of TCDD in the Sprague-Dawley rat. Objectives of this work were to characterize the influence of induction of CYP1A2 and adipose tissue mass fraction on the terminal elimination half-life (*t*_1/2_) of TCDD using this PBPK model. When the model assumes a fixed elimination of TCDD, *t*_1/2_ increases with dose, due to hepatic sequestration. Because experimental data indicate that the *t*_1/2_ of TCDD decreases with dose, the model was modified to include an inducible elimination rate. The PBPK model was then used to compare the *t*_1/2_ after an increase of adipose tissue mass fraction from 6.9 to 70%. The model suggests that at low exposures, increasing adipose tissue mass increases the terminal *t*_1/2_. However, at higher exposures, as CYP1A2 is induced, the relationship between adipose tissue mass and *t*_1/2_ reaches a plateau. This demonstrates that an inducible elimination rate is needed in a PBPK model in order to describe the pharmacokinetics of TCDD. At low exposures these models are more sensitive to parameters related to partitioning into adipose tissue.

2,3,7,8-Tetrachlorodibenzo-*p*-dioxin (TCDD) is a ubiquitous environmental contaminant that induces a wide spectrum of toxic responses (DeVito and Birnbaum 1995). A number of pharmacokinetic models for TCDD are available that incorporate various stages of sophistication, including classical pharmacokinetic models (Michalek et al. 2002; Pinsky and Lorber 1998), pseudophysiologic models (Aylward et al. 2005; Carrier et al. 1995a, 1995b), and more descriptive physiologically based pharmacokinetic (PBPK) models (Andersen et al. 1993, 1997; Emond et al. 2004; Kohn et al. 1996; Maruyama et al. 2002; Wang et al. 1997, 2000). Some epidemiologic studies use classical pharmacokinetic models to describe and quantify TCDD exposures (Crump et al. 2003; Flesch-Janys et al. 1996; Salvan et al. 2001; Steenland et al. 2001). The potential use of pharmacokinetic models in risk assessment to understand the relationship between exposure and tissue concentrations underscores the importance of developing biologically accurate models of the pharmacokinetics of TCDD and related chemicals.

The most recent pharmacokinetic models for TCDD have a number of similarities. All these models describe the distribution of TCDD as diffusion limited (Andersen et al. 1993, 1997; Aylward et al. 2005; Carrier et al. 1995a, 1995b; Emond et al. 2004; Kohn et al. 1996; Maruyama et al. 2002; Wang et al. 1997, 2000). In addition, most of these models include an inducible TCDD-binding protein in hepatic tissue. Experimental evidence demonstrates that this protein is cytochrome P450 1A2 (CYP1A2) (Diliberto et al. 1999; Staskal et al. 2005), whose expression is regulated by the aryl hydrocarbon receptor (AhR).

One major difference among these models is the description of the elimination of TCDD. Empirical models developed from epidemiologic data assume a first-order elimination rate with half-lives (*t*_1/2_) varying from 7 to 8.7 years (Aylward et al. 1996; Crump et al. 2003; Flesch-Janys et al. 1996; Steenland et al. 2001). The models of Wang et al. (2000), Maruyama et al. (2002), and Emond et al. (2004) also assume a constant hepatic clearance rate for TCDD. Andersen et al. (1993, 1997), Emond et al. (2005), and Kohn et al. (1996) assume that hepatic elimination of TCDD increases with dose. In the toxicokinetic model of van der Molen et al. (1998, 2000), the *t*_1/2_ of TCDD varies by body composition but not by dose. Aylward et al. (2005) extended the model of Carrier et al. (1995a, 1995b) by incorporating elimination due to lipid partitioning of TCDD from the blood into the large intestine based on published human data (Moser and McLachlan 2002). Despite these mechanistic differences, most models provide reasonable fits to the experimental data.

Dioxins are highly lipophilic and concentrate in adipose tissue. Recent studies suggest that body fat mass influences the elimination of TCDD (van der Molen et al. 1998, 2000). Michalek and Tripathi (1999) found that the TCDD *t*_1/2_ increases with body mass index (BMI) in humans. Increasing BMI alters the pharmacokinetics of lipophilic chemicals due to increased distribution into the adipose compartment and by altering xenobiotic metabolizing enzymes (Anzenbacher and Anzenbacherova 2001; Cheng and Morgan 2001).

TCDD metabolism, CYP1A2 induction, binding to CYP1A2, and BMI influence the elimination of TCDD (Olson et al. 1995). Thus, the objectives of this work were to characterize the influence of CYP1A2 induction and adipose tissue mass fraction on the terminal elimination *t*_1/2_ of TCDD using a rat PBPK model.

## Materials and Methods

**Figure 1:**
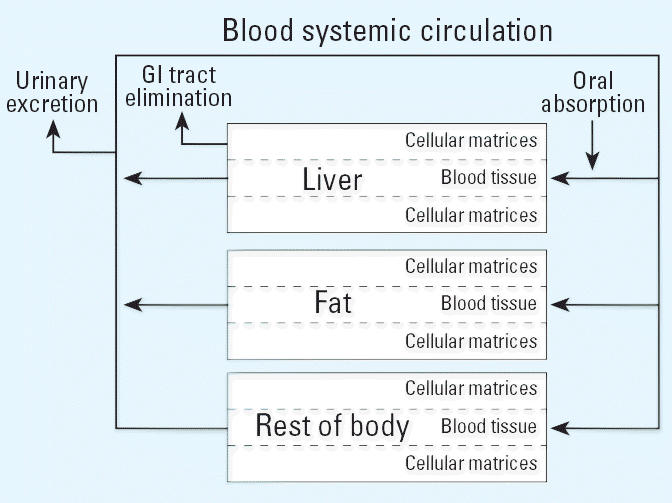
Conceptual representation of PBPK model for rat exposed to TCDD. GI, gastrointestinal.

This work is an extension of the TCDD PBPK model for Sprague-Dawley rats of Emond et al. (2004) that consists of four compartments: liver, fat, placenta (activated during gestation), and rest of the body (Figure 1). The systemic circulation interconnects each compartment. The present analysis focuses on nonpregnant animals, so the placental compartment was inactive. The liver compartment includes AhR-mediated induction of CYP1A2 and binding of TCDD to both the AhR and CYP1A2. Oral absorption and urinary and hepatic elimination were described, and constants were fit to the experimental data of Santostefano et al. (1998) as previously described (Emond et al. 2004). The elimination constant was optimized to incorporate hepatic metabolism, enterohepatic recirculation, and biliary elimination of TCDD. All physiological, biochemical, and physicochemical parameters used in this model are from Emond et al. (2004) (Table 1). The PBPK model was developed with algebraic and differential equations using ACSL software (Advanced Continuous Simulation Language; Aegis Corp., Huntsville, AL; see Appendix for equations). The original Emond et al. (2004) model is designated the fixed elimination model.

### Inducible elimination t_1/2_

A review of theexperimental estimates of the *t*_1/2_ of TCDD in rats suggests that the longest estimates of *t*_1/2_ are from studies using the lowest exposures (Table 2). This comparison includes data from different strains and sex of rats, and the influence of dose on the elimination of TCDD must be viewed cautiously. Most of the exposures were well above the median efffective dose (ED_50_) for enzyme induction (Santostefano et al. 1997). Data in mice and humans suggest that the elimination of TCDD is dose dependent (Abraham et al. 2002; Diliberto et al. 2001; Michalek et al. 2002).

The Emond et al. (2004) model was modified to include an inducible TCDD elimination that assumes the elimination increases proportionately to CYP1A2 induction:


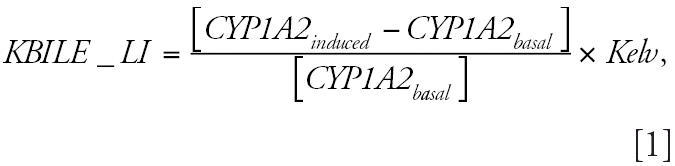


where *KBILE_LI* is the inducible elimination rate (hr^−1^), *CYP1A2_induced_* is the concentration of *CYP1A2* induced (nmol/mL), *CYP1A2_basal_* is the basal concentration of CYP1A2 (nmol/mL), and Kelv is the interspecies constant adjustment for the elimination rate (hr^−1^). In the model, *CYP1A2_induced_* is always greater than *CYP1A2_basal_*, and the difference between these two values is always positive. Kelv was optimized to the data of Santostefano et al. (1998) using ACSL Optimize (ACSL Math, version 2.1) using a maximization of the log-likelihood function (Steiner et al. 1990).

### Estimates of terminal elimination t_1/2_

The influence of KBILE_LI, BMI, CYP1A2 induction, and TCDD binding to CYP1A2 on the terminal elimination *t*_1/2_ of TCDD was examined using the fixed and inducible elimination models. The terminal elimination *t*_1 / 2_ of TCDD in blood was estimated between 300 and 900 hr from simulations of single oral exposures in a dose range from 10^−3^ to 10^3^ μg TCDD/kg using PK Solutions (version 2.0; Summit Research Solutions, Ashland, OH).

### Influence of CYP1A2 binding and BMI on the terminal elimination t_1/2_ of TCDD

The influence of CYP1A2 binding on the terminal elimination *t*_1/2_ of TCDD was examined by increasing the binding affinity of TCDD to CYP1A2 (KDLI2) from 0.04 to 10 ^6^ nmol/mL. An increase in KDLI2 to 10^6^ nmol/mL results in negligible TCDD binding to CYP1A2 and no hepatic sequestration while still allowing for the induction CYP1A2 and increased TCDD elimination.

The influence of BMI on the terminal elimination *t*_1/2_ of TCDD was examined by varying the size of the adipose tissue compartment from 6.9 to 70%. In order to maintain mass balance, the size of the rest of the body compartment decreases, which increases the size of the adipose compartment. Cardiac output and body weight (BW) remained constant as the adipose tissue compartment was increased.

### Sensitivity of parameters for a fixed or an inducible terminal elimination t_1/2_

Sensitivity analysis was performed on all parameters in the fixed and inducible elimination models at exposures of 0.001 and 10 μg TCDD/kg BW for which blood concentrations at 900 hr postexposure were compared. Exposures of 0.001 μg/kg result in negligible induction of CYP1A2, whereas 10 μg/kg exposures result in maximal induction of CYP1A2 in rats. The variation in the blood concentrations between optimized parameters and parameters (±10%) was calculated as follows:


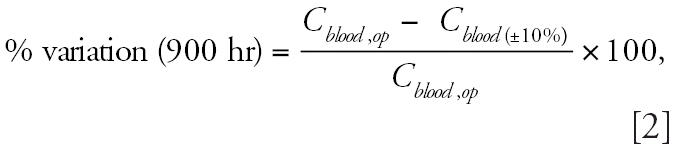


where *C_blood,op_* is the blood concentration obtained with the optimized parameter and *C_blood_*_(±10%)_ is the blood concentration obtained with the variation of the parameter.

## Results

### The influence of CYP1A2 induction and binding on the terminal elimination t_1/2_ of TCDD using a rat PBPK model

**Figure 2:**
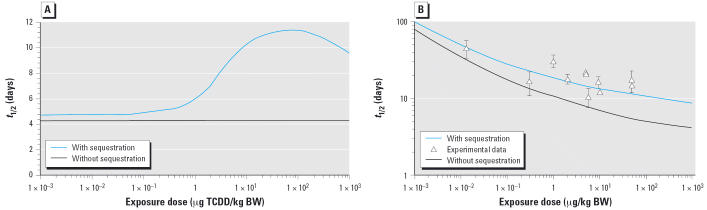
The relationship between terminal elimination *t*_1/2_ and dose using (*A*) a fixed elimination rate with and without CYP1A2 sequestration and (*B*) an inducible elimination rate with and without CYP1A2 sequestration. Triangles in *B* represent the TCDD *t*_1/2_ values determined experimentally (see Table 2).

The PBPK model for rats predicts that the terminal elimination *t*_1/2_ is constant at exposures of ≤0.1 μg/kg and increases to approximately 10 days as dose increases from 0.1 to approximately 100 μg/kg in the fixed elimination model. At exposures > 100 μg TCDD/kg BW, the terminal elimination *t*_1/2_ begins to decrease with exposure. Although the fixed elimination model provides adequate prediction of several experimental data sets (Emond et al. 2004; Wang et al. 1997), the terminal elimination *t*_1/2_ of TCDD is predicted to increase with dose. When the binding affinity to CYP1A2 is increased more than 7 orders of magnitude, hepatic sequestration does not occur and the model predicts a constant terminal elimination *t*_1/2_ at all exposures, suggesting that the predicted increases in *t*_1/2_ at high doses are due to hepatic sequestration mediated by CYP1A2 binding (Figure 2A).

The PBPK model was modified to describe the hepatic elimination rate (Kelv) as a function of CYP1A2 induction as described in Equation 1. The model was fit to the data of Santostefano et al. (1998). After optimization, Kelv was estimated as 0.15 hr^−1^. The model assumes a maximum 40-fold induction of CYP1A2, resulting in estimates of KBILE_LI from 0.06 to 2.46 hr^−1^ at exposures from 10^−3^ to 10^3^ μg/kg. Terminal elimination *t*_1/2_ estimates range from approximately 75 days at exposures of 10^−3^ μg/kg to approximately 10 days at the higher exposures. It should be noted that the experimental data range from 10 ^−2^ to 10^2^ μg TCDD/kg and that the model fits estimates of the *t*_1/2_ values relatively well, given the variability in the data (Figure 2B). Elimination of hepatic sequestration by CYP1A2 binding from the model decreases the terminal elimination *t*_1/2_ of TCDD at higher exposures.

**Figure 3:**
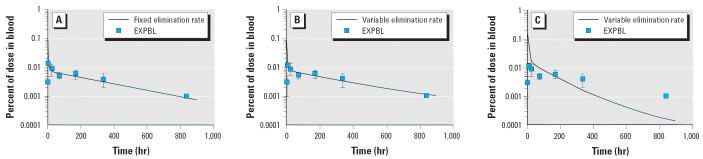
Comparisons of model predictions to experimental data using a fixed elimination rate model with hepatic sequestration (*A*) and an inducible elimination rate model with (*B*) and without (*C*) hepatic sequestration. EXBL, experimental blood levels. Model predictions were compared with the data of Santostefano et al. (1998), where female rats were exposed to a single oral dose of 10 μg of TCDD/ kg BW. Error bars are ± SD.

The use of an inducible elimination provides better fits to the experimental data of Santostefano et al. (1998) compared with the fixed elimination model (Figure 3A,B). These two simulations were performed at exposures of 10 μg TCDD/kg, which is a maximally CYP1A2-inducing dose of TCDD. The fixed elimination rate model was optimized at exposures near maximal induction; thus, at high exposures, the KBILE_LI used in the fixed model is not very different from the KBILE_LI derived in the inducible elimination model.

**Figure 4:**
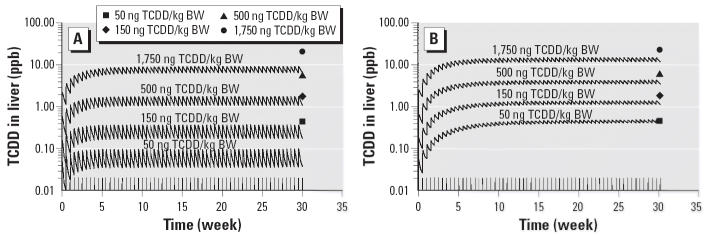
Simulation of hepatic TCDD concentrations (ppb) during a chronic exposure to TCDD at 50, 150, 500, or 1,750 ng TCDD/kg BW (Walker et al. 1999) using the fixed elimination rate model (*A*) or the inducible elimination rate model at (*B*) compared with the experimental data measured at the end of the exposures. Solid lines represent model simulations.

Differences between the two models also occur with simulations of the data from Walker et al. (1999), who exposed female Sprague-Dawley rats biweekly to 50, 150, 500, or 1,750 ng TCDD/kg and determined hepatic TCDD concentrations after 30 weeks of exposure. The fixed elimination rate model underestimated hepatic TCDD concentrations by 2- to 5-fold at the two highest doses and approximately an order of magnitude at the two lowest doses (Figure 4A). The inducible elimination model estimates the TCDD liver concentrations within the experimental data at the two lowest doses and underestimates the tissue concentrations at the two highest doses by less than a factor of 2 (Figure 4B).

### Influence of CYP1A2 sequestration on the terminal elimination t_1/2_ of TCDD using an inducible elimination model

The data from Santostefano et al. (1998) were used to examine the influence of CYP1A2 sequestration on the disposition of TCDD. A single dose of 10 μg TCDD/kg produces a maximal induction of CYP1A2. The inclusion of CYP1A2 sequestration in the model results in higher TCDD blood concentrations and provides good fits to the experimental data (Figure 3B). Removal of CYP1A2 sequestration from the model results in decreased TCDD blood concentrations and underestimates blood concentrations by more than an order of magnitude at the longer time points (Figure 3C).

### Influence of adipose tissue mass fraction on terminal elimination t_1/2_

**Figure 5:**
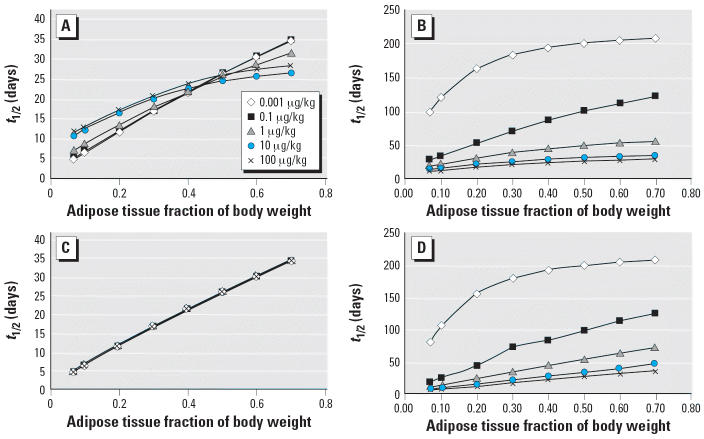
The influence of adipose tissue mass fraction on the predicted terminal elimination *t*_1/2_ after a single dose of 10 μg TCDD/kg. Simulations were performed with adipose tissue mass fraction ranging from 6.9 to 70% of body fat. Simulations using a fixed elimination model are presented with (*A*) and without (*B*) hepatic sequestration. Simulations using an inducible elimination model are presented with (C) and without (D) hepatic sequestrations.

In order to examine the role of adipose tissue in the terminal elimination *t*_1/2_ of TCDD, the adipose tissue compartment was varied from 6.9 to 70% in model simulations. In the fixed elimination rate model, there is a linear relationship between increases in the size of the adipose tissue compartment and the *t*_1/2_ of TCDD at low exposures (Figure 5A). The influence of the size of the adipose tissue compartment diminishes as TCDD exposure increases (Figure 5A). When the hepatic sequestration is removed from the model, the relationship between increases in the size of the adipose tissue compartment and *t*_1/2_ is linear and independent of TCDD exposure (Figure 5B). Using the inducible elimination rate model, the terminal elimination *t*_1/2_ increases with dose for models with and without hepatic sequestration (Figure 5C,D).

### Sensitivity of parameters for a fixed or an inducible elimination t_1/2_

**Figure 6:**
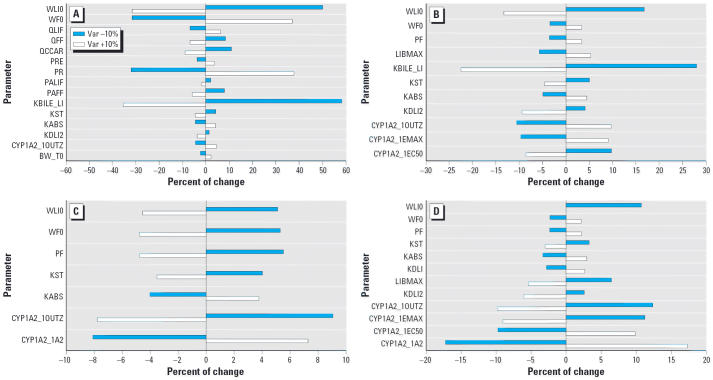
Sensitivity analysis was performed on the fixed elimination rate model (*A* and *B*) and the inducible elimination rate model (*C* and *D*). The analysis was performed at 0.001 μg/kg (*A* and *C*) and at 10 μg/kg (*B* and *D*). Abbreviations: BW_T0, body weight at time zero (other parameter symbols are defined in Table 1); var, variation. This sensitivity recorded the percentage of variation (≥ 2%) of TCDD concentrations in the blood compartment when parameters were varied by ±10%.

Sensitivity analysis was performed on all parameters in the fixed and inducible elimination models for acute exposures of 0.001 μg/kg and 10 μg/kg. To simplify the presentation of the analysis, only parameters that resulted in a normalized sensitivity coefficient of ±2.0% are discussed. In the fixed elimination model, 15 parameters have normalized sensitivity coefficients greater than ±2% at the low dose, and 11 parameters at the high dose (Figure 6A,B). In the inducible elimination model, the sensitivity analysis indicates that 7 parameters have normalized sensitivity coefficients greater than ± 2% at the low dose, and 12 parameters at the high dose (Figure 6C,D). Six parameters were sensitive for both exposures and models. Two of the common parameters were related to absorption [gastric nonabsorption constant (KST) and intestinal absorption rate (KABS)], and two represent liver and adipose tissue volume fraction (WLI0 and WF0, respectively). Both models and exposure levels are sensitive to the fat partition coefficient (PF) and the degradation rate for CYP1A2 (CYP1A2_1OUTZ). The low-dose exposure in the fixed elimination model is uniquely sensitive to parameters related to the distribution of TCDD such as cardiac output, BW, blood flow, and partitioning to liver and fat. The high-dose exposures in both models are sensitive to parameters related to CYP1A2 induction, such as maximal induction of CYP1A2 (CYP1A2_1EMAX), dissociation constant during induction (CYP1A2_1EC50), and AhR binding capacity in hepatic tissue (LIBMAX). Both low- and high-dose exposures in the variable elimination model are uniquely sensitive to the basal CYP1A2 expression (CYP1A2_1A2).

## Discussion

The elimination of TCDD in mammals depends on diffusion into and out of adipose tissue, metabolism, hepatic sequestration, and hepatic elimination rate. The present study examined the relationship between these factors using a PBPK model. The Emond et al. (2004) PBPK model indicates that the *t*_1/2_ of TCDD increases with increasing exposure, which is inconsistent with some experimental (Table 2) and human data suggesting that the *t*_1/2_ decreases with exposure. Modification of the Emond et al. (2004) model to include inducible hepatic elimination better fits the experimental data of Santostefano et al. (1998) and Walker et al. (1999). With an inducible elimination, the *t*_1/2_ of TCDD varies from approximately 75 days to 10 days after exposures ranging from 10^−3^ to 10^3^ μg TCDD/kg, respectively.

The inducible elimination model describes the elimination rate as a function of CYP1A2 induction. TCDD induces several xenobiotic-metabolizing enzymes, including CYP1A1, CYP1A2, and CYP1B1. The role of these enzymes in the metabolism of TCDD is not clear because of limited data on *in vitro* and *in vivo* metabolism of TCDD. The role of CYP1A in the metabolism of TCDD is inferred from *in vitro* metabolism of lesser chlorinated dioxins or 2,3,7,8-tetrachloro-dibenzofuran (Olson et al. 1995; Shinkyo et al. 2003; Tai et al. 1993). *In vivo* studies examining biliary elimination of radioactivity in rats treated with [H^3^]TCDD have not been able to demonstrate inducible elimination of TCDD-derived radioactivity (Kedderis et al. 1991). Poiger and Schlatter (1985) observed a doubling of the biliary elimination of TCDD in dogs pretreated with TCDD, indicating a role for CYP1A in the elimination of TCDD.

One of the problems in quantifying the role of CYP1A2 in the metabolism and elimination of TCDD is that CYP1A2 both binds and metabolizes TCDD. TCDD inhibits rat and human CYP1A2 activity (Staskal et al. 2005). In *CYP1A2* knockout mice, there is no hepatic sequestration of TCDD, adipose tissue TCDD concentrations are higher, and the levels of metabolites in urine and feces are lower compared with wild-type mice (Diliberto et al. 1999; Hakk and Diliberto 2002). These studies as a whole indicate that CYP1A2 and other CYPs are involved in the metabolism and elimination of TCDD.

The inducible elimination model predicts that the terminal elimination *t*_1/2_ of TCDD increases approximately 10-fold, whereas the elimination rate from hepatic tissue increases > 40-fold. One possible explanation for this discrepancy is that diffusion into and out of adipose tissue is the rate-limiting step in the elimination of TCDD at low exposures and that metabolic elimination is the rate-limiting step at high exposures. The model predicts that estimates of the *t*_1/2_ are more sensitive to changes in BMI at low exposures than at higher exposures. When significant induction of CYP1A2 occurs, there is an increase in hepatic sequestration and elimination, which dampens the effects of changes in BMI. These observations are consistent with experimental data in the CYP1A2 knockout mouse (Diliberto et al. 1999; Hakk and Diliberto 2002).

Pharmacokinetic models for TCDD describe its elimination in a variety of ways. The Andersen et al. (1993) model describes induction as a function of receptor occupancy multiplied by a species-specific adjustment factor designated as “fold.” For rats, this parameter was assigned a value of 1 (Andersen et al. 1993), resulting in a doubling of TCDD metabolism over the basal rate. Carrier et al. (1995a, 1995b) used a simple first-order elimination process that is a function of total hepatic TCDD concentrations. In the Carrier et al. model, hepatic concentrations increase with dose in a nonlinear manner because of hepatic sequestration. As the fraction of TCDD in the liver increases from 15 to 70%, there is a 5-fold maximum induction of the elimination rate in rats. For humans, the model estimates that the fraction of TCDD in the liver ranges from 1 to 70%, resulting in an approximately 70-fold induction of TCDD elimination at high exposures (Carrier et al. 1995a, 1995b). The Kohn et al. (1996) model uses Hill kinetics to describe the elimination of TCDD with a Hill exponent of greater than unity. The Kohn et al. (1996) model also includes a biliary elimination of TCDD that is a function of a TCDD-induced hepatic lytic rate (hepatotoxicity) and a measure of cumulative exposure. In the Kohn et al. (1996) model, once the cells die, the TCDD is eliminated through the bile into the gut with a linear rate, implying diffusion. The difference in the description of the elimination pathways between these models is based on the lack of known metabolic processes involved in the elimination of TCDD.

TCDD metabolism may not be the only route of elimination of TCDD. Aylward et al. (2005) extended the Carrier et al. (1995a, 1995b) model to include lipid partitioning of TCDD from circulation into the large intestine followed by fecal elimination, based on the work of Moser and McLachlan (2001). Although this pathway is not described in the present model, the elimination of TCDD from the blood into the intestines is indirectly accounted for in the optimized elimination rate. Our ability to discriminate between these different modeling approaches is diminished by our lack of understanding of the enzymes metabolizing TCDD and the role of lipid partitioning and hepato-toxicity in the pharmacokinetics of TCDD.

The dose-dependent elimination of dioxins can influence exposure assessments in epidemiologic studies assessing the potential adverse health effects of dioxins. Several of the epidemiologic studies examine the relationship between dioxin exposure and adverse health effects. Some of these analyses use a first-order elimination rate from present measured body burdens to back-calculate TCDD body burdens at the initial exposure (Crump et al. 2003; Steenland et al. 2001). Aylward et al. (2005) and Emond et al. (2005) suggest that using a pharmacokinetic model with dose-dependent elimination results in nonlinear relationships between measured body burdens and predicted peak body burdens. Applying PBPK models that include inducible elimination rates to the epidemiologic data may result in quantitatively different relationships between exposure and adverse health effects observed in these studies.

## Figures and Tables

**Table 1:** Physiologic parameters used in the PBPK models for rat.^a^

Parameter description	Symbol	Value
Body weight (g)	BW	250
Cardiac output (mL/hr/kg)	QCCAR	311.4
Tissue volumes (fraction of BW)		
Liver	WLI0	0.036
Fat	WF0	0.069
Rest of the body	WRE0	0.729
Blood	WB0	0.076
Tissues blood volumes		
Liver (fraction of liver)	WLIB0	0.266
Fat (fraction of fat)	WFB0	0.050
Rest of the body (fraction of rest of the body)	WREB0	0.030
Tissue blood flows (fraction of cardiac output)		
Liver	QLIF	0.183
Fat	QFF	0.069
Rest of the body	QREF	0.748
Tissue permeability (fraction of tissue blood flow)	
Liver	PALIF	0.3500
Fat	PAFF	0.0910
Rest of the body	PAREF	0.0298
Partition coefficient		
Liver	PLI	6
Fat	PF	100
Rest of the body	PRE	1.5
Metabolism constants		
Urinary clearance elimination (mL/hr)	CLURI	0.01
Liver (biliary elimination and metabolism; hr^−1^)	KBILE_LI	Inducible^b^
Interspecies constant (hr^−1^)	Kelv	0.15^c^
AhR		
Affinity constant in liver (nmol/mL)	KDLI	0.0001
Binding capacity in liver (nmol/mL)	LIBMAX	0.00035
CYP1A2 induction parameters		
Dissociation constant CYP1A2 (nmol/mL)	KDLI2	0.04
Degradation process CYP1A2 (nmol/mL)	CYP1A2_1OUTZ	1.6
Dissociation constant during induction (nmol/mL)	CYP1A2_1EC50	0.3
Basal concentration of CYP1A2 (nmol/mL)	CYP1A2_1A2	1.6
First-order rate for degradation (hr^−1^)	CYP1A2_1KOUT	0.1
Time delay before induction process (hr)	CYP1A2_1TAU	0.25
Maximal induction of CYP1A2 (unitless)	CYP1A2_1EMAX	600
Other constant		
Oral absorption constant (hr^−1^)	KABS	0.48
Gastric nonabsorption constant (hr^−1^)	KST	0.36

a From Emond et al. (2004), except as specified.

b In the fixed elimination model this value is 2.2 hr^−1^ as presented by Emond et al. (2004). In the inducible elimination model this parameter varies with exposure as described in Equation 1.

c Formal optimization followed the visual fitting.

**Table 2:** Relation between dose and *t*_1/2_ calculated in experimental data in rats.^a^

Strain	Sex	Dose (μg/kg)	*t*_1/2_ ± SD (days)	Reference
Wistar	F	0.3	16.6 ± 5.7	Abraham et al. 1988
Wistar	M	0.01	45.2 ± 11.4	Lakshmanan et al. 1986
Wistar	M	5.0	21.9	Pohjanvirta et al. 1990
Long Evans	M	5.0	20.8	Pohjanvirta et al. 1990
Long Evans	M	2	18.2 ± 2.6	Viluksela et al. 1996
Long Evans	M	5.6	10.5 ± 2.8	Viluksela et al. 1996
Sprague-Dawley	F	10	12	Wang et al. 1997
Sprague-Dawley	M	1	31 ± 6	Rose et al. 1976
Sprague-Dawley	M	9.25	16.3 ± 3	Weber et al. 1993
Sprague-Dawley	M	50	17.4 ± 5.4	Piper et al. 1973
Sprague-Dawley	M	50	14.5 ± 0.5	Allen et al. 1975

Abbreviations: F, female, M, male.

a All experimental paradigms used a single exposure.

## Appendix. Equations used in the PBPK model for adult rat.^a^

**Table:**

**Body weight growth with age**

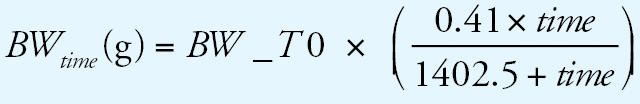

**Cardiac output**

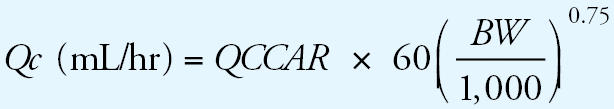

A factor of 60 corresponds to the conversion of minutes to hours, and 1,000 is conversion of BW from grams to kilograms.
**Blood compartment**

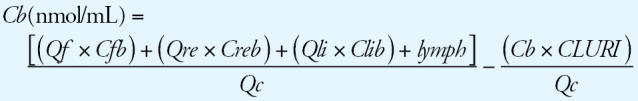

**Tissue compartment (fat, rest of the body)**
Tissue blood subcompartment

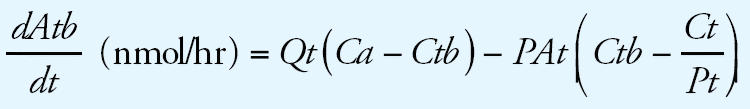

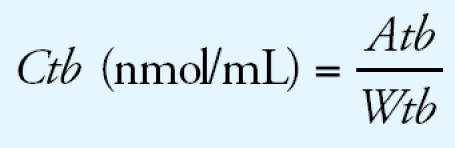

Tissue cellular matrices

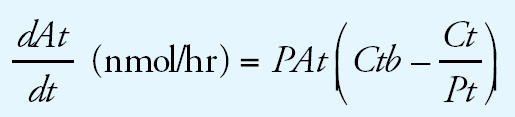

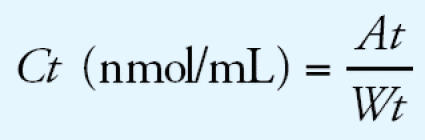

**Liver tissue compartment**
Tissue blood subcompartment

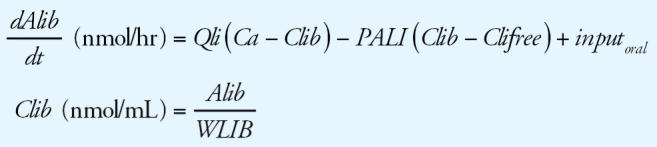

Tissue cellular matrices

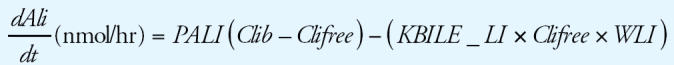

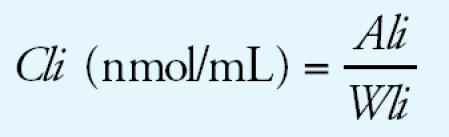

**Free TCDD concentration in liver**

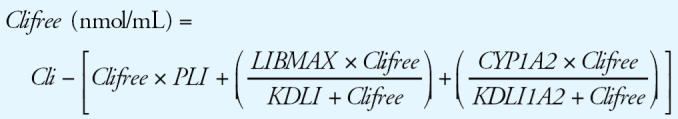

**Concentration bound to AhR in hepatic tissue**

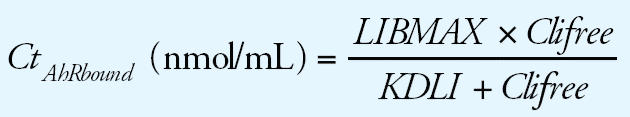

All others induction processes and equations have been described and presented by Wang et al. (1997).
**Gastrointestinal absorption and distribution of TCDD to the portal lymphatic circulation**
Amount of TCDD remaining in lumen cavity

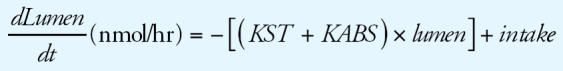

Lumen is the amount of TCDD remaining in the GI tract (nmol); intake is the rate of intake of TCDD during a subchronic exposure (nmol/hr).
Amount of TCDD eliminated in the feces

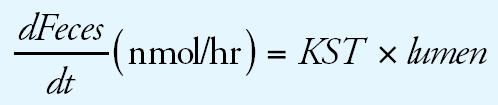

Absorption rate of TCDD to the blood via the lymphatic circulation

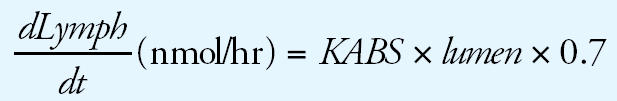

Absorption rate of TCDD by the liver via by portal circulation

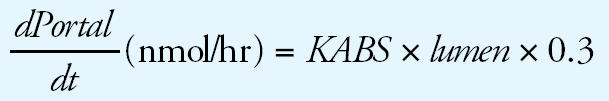

Abbreviations and parameter symbols: *Ali*, amount of chemical in liver cellular matrice subcompartment; *Alib*, amount of chemical in liver in hepatic tissue blood subcompartment; *At*, amount of chemical in tissue cellular matrice subcompartment; *Atb*, amount of chemical in tissue blood subcompartment; *Ca*, arterial concentration; *Cb*, blood systemic venous concentration; *Cfb*, adipose tissue blood subcompartment concentration; *CLI*, liver blood subcompartment concentration; *Clib*, liver tissue blood subcompartment concentration; *Clifree*, free chemical concentration in liver compartment; *Creb*, rest of the body blood subcompartment concentration; *Ct*, tissue concentration in cellular matrice; *Ctb*, tissue blood subcompartment concentration; *dAli,* variation of the amount of chemical in hepatic compartment with time; *dAlib/dt*, variation of the amount of chemical in hepatic blood compartment with time; *dAtb/dt*, variation of the amount of chemical in blood subcompartment with time; *input**_oral_*, rate of oral chemicals intakes; *PALI*, liver tissue permeability (*PALIF* × *QLI*); *PAt*, tissue permeability (*PATF* (tissue permeability) × *Qt*); *Pt*, partition coefficient in tissue compartment; *Qc*, cardiac output; *Qf*, adipose tissue blood flow (*QFF* × *Qc*); *Qli*, liver tissue blood flow (*QLIF* × *Qc*); *Qre*, rest of the body blood flow (*QREF* × *Qc*); *Qt*, blood flow in tissue compartment; *WLI*, volume of liver cellular matrice tissue subcompartment; *Wt*, volume of cellular matrice tissue subcompartment; *Wtb*, volume of tissue blood subcompartment.

a For more information refer to Emond et al. (2004).

## References

[b1-ehp0114-001394] Abraham K, Geusau A, Tosun Y, Helge H, Bauer S, Brockmoller J (2002). Severe 2,3,7,8-tetrachlorodibenzo-*p*-dioxin (TCDD) intoxication: insights into the measurement of hepatic cytochrome P450 1A2 induction. Clin Pharmacol Ther.

[b2-ehp0114-001394] Abraham K, Krowke R, Neubert D (1988). Pharmacokinetics and biological activity of 2,3,7,8-tetrachlorodibenzo-*p*-dioxin. 1: Dose-dependent tissue distribution and induction of hepatic ethoxyresorufin *O*-deethylase in rats following a single injection. Arch Toxicol.

[b3-ehp0114-001394] Allen JR, Van Miller JP, Norback DH (1975). Tissue distribution, excretion and biological effects of [^14^C]tetrachlorodibenzo-*p*-dioxin in rats. Food Cosmet Toxicol.

[b4-ehp0114-001394] Andersen ME, Birnbaum LS, Barton HA, Eklund C (1997). Regional hepatic CTP1A1 and CYP1A2 induction with 2,3,7,8-tetra-chlorodibenzo-*p*-dioxin evaluated with a multicompartment geometric model of hepatic zonation. Toxicol Appl Pharmacol.

[b5-ehp0114-001394] Andersen ME, Mills JJ, Gargas ML, Kedderis L, Birnbaum LS, Neubert D (1993). Modeling receptor-mediated processes with dioxin: implications for pharmacokinetics and risk assessment. Risk Anal.

[b6-ehp0114-001394] Anzenbacher P, Anzenbacherova E (2001). Cytochromes P450 and metabolism of xenobiotics. Cell Mol Life Sci.

[b7-ehp0114-001394] Aylward LL, Brunet RC, Carrier G, Hays SM, Cushing CA, Needham LL (2005). Concentration-dependent TCDD elimination kinetics in humans: toxicokinetic modeling for moderately to highly exposed adults from Seveso, Italy, and Vienna, Austria, and impact on dose estimates for the NIOSH cohort. J Expo Anal Environ Epidemiol.

[b8-ehp0114-001394] Aylward LL, Hays SM, Karch NJ, Paustenbach DJ (1996). Relative suceptibility of animals and humans to the cancer hazard posed by 2,3,7,8-tetrachlorodibenzo-*p*-dioxin using internal measures. Environ Sci Technol.

[b9-ehp0114-001394] Carrier G, Brunet RC, Brodeur J (1995a). Modeling of the toxicokinetics of polychlorinated dibenzo-*p*-dioxins and dibenzofuranes in mammalians, including humans. II: Kinetics of absorption and disposition of PCDDs/PCDFs. Toxicol Appl Pharmacol.

[b10-ehp0114-001394] Carrier G, Brunet RC, Brodeur J (1995b). Modeling of the toxicokinetics of polychlorinated dibenzo-*p*-dioxins and dibenzofurans in mammalians, including humans. Toxicol Appl Pharmacol.

[b11-ehp0114-001394] Cheng PY, Morgan ET (2001). Hepatic cytochrome P450 regulation in disease states. Curr Drug Metab.

[b12-ehp0114-001394] Crump KS, Canady R, Kogevinas M (2003). Meta-analysis of dioxin cancer dose response for three occupational cohorts. Environ Health Perspect.

[b13-ehp0114-001394] DeVito MJ, Birnbaum LS (1995). Dioxins: model chemicals for assessing receptor-mediated toxicity. Toxicology.

[b14-ehp0114-001394] Diliberto JJ, Burgin DE, Birnbaum LS (1999). Effects of CYP1A2 on disposition of 2,3,7,8-tetrachlorodibenzo-*p*-dioxin, 2,3,4,7,8-pentachlorodibenzofuran, and 2,2’,4,4’,5,5’-hexachloro-biphenyl in CYP1A2 knockout and parental (C57BL/6N and 129/Sv) strains of mice. Toxicol Appl Pharmacol.

[b15-ehp0114-001394] Diliberto JJ, DeVito MJ, Ross DG, Birnbaum LS (2001). Subchronic exposure of [3H]- 2,3,7,8-tetrachlorodibenzo-*p*-dioxin (TCDD) in female B6C3F1 mice: relationship of steady-state levels to disposition and metabolism. Toxicol Sci.

[b16-ehp0114-001394] Emond C, Birnbaum LS, DeVito M (2004). Physiologically based pharmacokinetic model for developmental exposures to TCDD in the rat. Toxicol Sci.

[b17-ehp0114-001394] Emond C, Michalek JE, Birnbaum LS, Devito MJ (2005). Comparison of the use of a physiologically based pharmacokinetic model and a classical pharmacokinetic model for dioxin exposure assessments. Environ Health Perspect.

[b18-ehp0114-001394] Flesch-Janys D, Becher H, Gurn P, Jung D, Konietzko J, Manz A (1996). Elimination of polychlorinated dibenzo-*p*-dioxins and dibenzofurans in occupationally exposed persons. J Toxicol Environ Health.

[b19-ehp0114-001394] Hakk H, Diliberto JJ (2002). Comparison of overall metabolism of 2,3,7,8-TCDD in CYP1A2 (−/−) knockout and C57BL/6N parental strains on mice. Organohalogen Compounds.

[b20-ehp0114-001394] Kedderis LB, Diliberto JJ, Linko P, Goldstein JA, Birnbaum LS (1991). Disposition of 2,3,7,8-tetrabromodibenzo-*p*-dioxin and 2,3,7,8,-tetrachlorodibenzo-*p*-dioxin in the rat: biliary excretion and induction of cytochrome CYP1A1 and CYP1A2. Toxicol Appl Pharmacol.

[b21-ehp0114-001394] Kohn MC, Sewall CH, Lucier GW, Portier CJ (1996). A mechanistic model of effects of dioxin on thyroid hormones in the rat. Toxicol Appl Pharmacol.

[b22-ehp0114-001394] Lakshmanan MR, Campbell BS, Chirtel SJ, Ekarohita N, Ezekiel M (1986). Studies on the mechanism of absorption and distribution of 2,3,7,8-tetrachlorodibenzo-*p*-dioxin in the rat. J Pharmacol Exp Ther.

[b23-ehp0114-001394] Maruyama W, Yoshida K, Tanaka T, Nakanishi J (2002). Possible range of dioxin concentration in human tissues: simulation with a physiologically based model. J Toxicol Environ Health A.

[b24-ehp0114-001394] Michalek JE, Pirkle JL, Needham LL, Patterson DG, Caudill SP, Tripathi RC (2002). Pharmacokinetics of 2,3,7,8-tetra-chlorodibenzo-*p*-dioxin in Seveso adults and veterans of operation Ranch Hand. J Expo Anal Environ Epidemiol.

[b25-ehp0114-001394] Michalek JE, Tripathi RC (1999). Pharmacokinetics of TCDD in veterans of Operation Ranch Hand: 15-year follow-up. J Toxicol Environ Health A.

[b26-ehp0114-001394] Moser GA, McLachlan MS (2002). Partitioning of polychlorinated biphenyls and hexachlorobenzene into human faeces. Chemosphere.

[b27-ehp0114-001394] Olson JR, McGarrigle BP, Gigliotti PJ, Kumar S, McReynolds JH (1995). Hepatic uptake and metabolism of 2,3,7,8-tetra-chlorodibenzo-*p*-dioxin and 2,3,7,8-tetrachlorodibenzofuran. Fundam Appl Toxicol.

[b28-ehp0114-001394] Pinsky PF, Lorber MN (1998). A model to evaluate past exposure to 2,3,7,8-TCDD. J Expo Anal Environ Epidemiol.

[b29-ehp0114-001394] Piper WN, Rose JQ, Gehring PJ (1973). Excretion and tissue distribution of 2,3,7,8-tetrachlorodibenzo-*p*-dioxin in the rat. Environ Health Perspect.

[b30-ehp0114-001394] Pohjanvirta R, Vartiainen T, Uusi-Rauva A, Monkkonen J, Tuomisto J (1990). Tissue distribution, metabolism, and excretion of ^14^C-TCDD in a TCDD-susceptible and a TCDD-resistant rat strain. Pharmacol Toxicol.

[b31-ehp0114-001394] Poiger H, Schlatter C (1985). Influence of phenobarbital and TCDD on the hepatic metabolism of TCDD in the dog. Experientia.

[b32-ehp0114-001394] Rose JQ, Ramsey JC, Wentzler TH, Hummel RA, Gehring PJ (1976). The fate of 2,3,7,8-tetrachlorodibenzo-*p*-dioxin following single and repeated oral doses to the rat. Toxicol Appl Pharmacol.

[b33-ehp0114-001394] Salvan A, Thomaseth K, Bortot P, Sartori N (2001). Use of a toxico-kinetic model in the analysis of cancer mortality in relation to the estimated absorbed dose of dioxin (2,3,7,8-tetra-chlorodibenzo-*p*-dioxin, TCDD). Sci Total Environ.

[b34-ehp0114-001394] Santostefano MJ, Ross DG, Savas U, Jefcoate CR, Birnbaum LS (1997). Differential time-course and dose-response relationships of TCDD-induced CYP1B1, CYP1A1, and CYP1A2 proteins in rats. Biochem Biophys Res Commun.

[b35-ehp0114-001394] Santostefano MJ, Wang F, Richardson VM, Ross DG, Devito MJ, Birnbaum LS (1998). A pharmacodynamic analysis of TCDD-induced cytochrome P450 gene expression in multiple tissues: dose- and time-dependent effects. Toxicol Appl Pharmacol.

[b36-ehp0114-001394] Shinkyo R, Sakaki T, Ohta M, Inouye K (2003). Metabolic pathways of dioxin by CYP1A1: species difference between rat and human CYP1A subfamily in the metabolism of dioxins. Arch Biochem Biophys.

[b37-ehp0114-001394] Staskal DF, Diliberto JJ, Devito MJ, Birnbaum LS (2005). Inhibition of human and rat CYP1A2 by TCDD and dioxin-like chemicals. Toxicol Sci 2005.

[b38-ehp0114-001394] Steenland K, Deddens J, Piacitelli L (2001). Risk Assessment for 2,3,7,8-tetrachlorodibenzo-*p*-dioxin (TCDD) based on an epidemiologic study. Am J Epidemiol.

[b39-ehp0114-001394] Steiner EC Rey TD McCroskey PS 1990. Reference Guide for Simusolv. Midland, MI:Dow Chemical Co.

[b40-ehp0114-001394] Tai HL, McReynolds JH, Goldstein JA, Eugster HP, Sengstag C, Alworth WL (1993). Cytochrome P4501A1 mediates the metabolism of 2,3,7,8-tetrachlorodibenzofuran in the rat and human. Toxicol Appl Pharmacol.

[b41-ehp0114-001394] van der Molen GW, Kooijman SA, Michalek JE, Slob W (1998). The estimation of elimination rates of persistent compounds: a re-analysis of 2,3,7,8-tetrachlorodibenzo-*p*-dioxin levels in Vietnam veterans. Chemosphere.

[b42-ehp0114-001394] van der Molen GW, Kooijman BA, Wittsiepe J, Schrey P, Flesch-Janys D, Slob W (2000). Estimation of dioxin and furan elimination rates with a pharmacokinetic model. J Expo Anal Environ Epidemiol.

[b43-ehp0114-001394] Viluksela M, Duong TV, Stahl BU, Li X, Tuomisto J, Rozman KK (1996). Toxicokinetics of 2,3,7,8-tetrachlorodibenzo-*p*-dioxin (TCDD) in two substrains of male Long-Evans rats after intravenous injection. Fundam Appl Toxicol.

[b44-ehp0114-001394] Walker NJ, Portier CJ, Lax SF, Crofts FG, Li Y, Lucier GW (1999). Characterization of the dose-response of CYP1B1, CYP1A1, and CYP1A2 in the liver of female Sprague-Dawley rats following chronic exposure to 2,3,7,8-tetrachlorodibenzo-*p*-dioxin. Toxicol Appl Pharmacol.

[b45-ehp0114-001394] Wang X, Santostefano MJ, Devito MJ, Birnbaum LS (2000). Extrapolation of a PBPK model for dioxins across dosage regimen, gender, strain, and species. Toxicol Sci.

[b46-ehp0114-001394] Wang X, Santostefano MJ, Evans MV, Richardson VM, Diliberto JJ, Birnbaum LS (1997). Determination of parameters responsible for pharmacokinetic behavior of TCDD in female Sprague-Dawley rats. Toxicol Appl Pharmacol.

[b47-ehp0114-001394] Weber LW, Ernst SW, Stahl BU, Rozman K (1993). Tissue distribution and toxicokinetics of 2,3,7,8-tetrachlorodibenzo-*p*-dioxin in rats after intravenous injection. Fundam Appl Toxicol.

